# Difficulties of Diagnosis

**Published:** 1858-08

**Authors:** R. J. Perry

**Affiliations:** Vienna, Ala.


### ARTICLE II.

*Difficulties of Diagnosis.* By Dr. R. J. PERRY, Vienna, Ala.

*Mess. Editors of the Atlanta Med. & Surg. Journal*—

I observed in the last number of your Journal, (April No., 1858,) a very interesting article, from one of your contributors, upon "The Difficulties of Diagnosis in Pneumonia;" and to my mind, he proves, conclusively, that even in the absence of several of the most prominent symptoms that are considered as indicative of this disease, it is often really found to exist. My mind was strongly impressed by this statement, and I have long been well convinced, that practitioners are too apt, in attempting an exhibition of their superior skill by hastily pronouncing what a disease is, or what it is not, to commit the most serious blunders. How often is it the case that we are told that Doctor A. or B. is attending a large number of patients who are laboring under some serious disease, when a thorough investigation of their condition proves that such is not the fact, thus jeopardizing their professional skill, or character as gentlemen, or, perhaps, both. I am, however, disposed to believe that such errors should be attributed mainly to haste and carelessness, rather than to the want of skill or evil design on the part of physicians. Again, there is another habit too common with practitioners, and that is pronouncing in the presence of their patients the name of the disease with which they are afflicted; and more especially so, if that name is likely to excite or alarm them.

In reviewing my *Note Book*, of important cases, my attention has been called to one, the diagnosis of which perplexed me much, and as it is interesting in other respects, I am disposed briefly to report it to the profession.

I was called on the night of the 23d of November, 1856, to see Mr. T———, a ferryman, of Vienna, aged 26 years, and found him complaining of pain in the left side, and saying that he could not get a *long breath*—had had a chill the previous night; pulse 110; some headache; and was expectorating a dark colored or brownish sputa. On applying my ear to the left side of the thorax, and over the seat of the pain, I either heard, or imagined I heard, the sound common to the incipient stage of pulmonic inflammation—minute crepitation—and pronounced his disease to be pneumonitis, and treated him accordingly. In three or four days he was able to set up, and appeared to be convalescent. On the 30th day of the same month, I left home, to be absent for a few days, and on the night of that day, the same symptoms as were present at the outset of the attack, returned, and Doctor P. was called to see him. He also considered the disease to be pneumonia, and of course prescribed accordingly. Again, in a few days he began to improve, and by the 12th of December was able to walk about town. That night, and for the third time, he was again seized with similar symptoms as at first, except that he only complained of pain in the side while coughing, and was inclined to sleep almost incessantly. The same kind of bloody or rust colored sputa was discharged; tongue furred, with red tip and edges; pulse 120; and complained of being unable to use his arms freely, or to turn over in bed; skin dry and rough; eyes dull and watery. Tenderness and dull pain in the left lumbar region. I now suspected the existence of an abscess in the left lung; cupped and blistered the left side; and administered, successively, calomel and Dover's powder, antimony, veratrum, &c.

*On the* 13*th*.—Symptoms the same; no action from the calomel; pulse 120; ordered oil at night.

14*th*.—Symptoms the same; ordered antimony in larger proportions than before.

15*th*.—Bowels freely moved last night; pulse 110; continued the veratrum.

16*th*.—Patient worse; severe pain in the left arm, and a red streak along the course of the brachial artery.

17*th*.—Cough not so troublesome; respiration improved; pulse 100; bowels moved twice during the previous night; drew 8 ozs. of blood; directed an emollient poultice to be applied to the arm; and ordered $\frac{1}{4}$th of a grain of morphine, with 2 grains of calomel, every three hours; and continued the veratrum.

18*th*.—Symptoms worse; pain in both arms; had slept none through the night; tongue still coated; pulse the same; pain in the region of the heart. Gave half a grain of morphine, and requested a consultation with Doctor M., of Fairfield, who, upon receiving a history of the case, hesitated whether to consider the disease pneumonia or dengue. He suggested Lugol's solution of iodine, and the 1-12th of a grain of strychnine every six hours, and a sufficiency of opium to quiet the patient.

19*th*, 20*th*, 21*st*.—Symptoms nearly the same, except when under the influence of potent anodynes.

From this time until the 30th of the month, there was a gradual but slight abatement of the symptoms. Tongue pretty well cleaned off; pulse 90; pains shifting from one extremity to another, and then to the head, neck, trunk, &c. Nor will I further protract an account of the varied and numerous symptoms that, from time to time, presented themselves; nor detail the diversified treatment employed by myself and others; suffice it to say, that he was finally reduced to the condition of almost a living skeleton, and was for sometime unable to move either his hands or feet.

With some slight degree of improvement in general, he reached the 13th of February, 1857, when, during a violent fit of coughing, a greenish looking pus or matter, of a very bitter taste, began to discharge copiously from the mouth, evidently the result of a ruptured abscess. The quantity discharged was enormous, and continued to escape for weeks. The pains, and other unpleasant symptoms, gradually subsided under appropriate treatment. He, by slow degrees, gained his strength and health, and is now as well as before he was attacked.

I should have mentioned, that prior to his first illness, he had been subject to occasional chills, and was predisposed to anasarca, and some cough. Efforts had been made to produce ptyalism, but they were unsuccessful. The questions at issue are, was the abscess a cause or consequence of the pneumonic, hepatic and hydropic disturbances, separate or combined, and was it hepatic or pulmonic. That the primary lesion was in the liver, I am strongly inclined to believe. If so, it is obvious that we were essentially mistaken in our diagnosis;* and now in conclusion, allow me to remark, that the object of this communication is mainly to awaken the profession, and more especially the junior members of it, to the importance of "Difficulty in Diagnosis."

* While writing this report, Mr. T. informs me that from the time he commenced *swelling up*, some six weeks prior to my first being called in to prescribe for him, he had a dull aching or uneasiness in his left side, which became painful and swollen, and remained so until the time of my first visit.

